# Assessing journal author guidelines for systematic reviews and meta-analyses: findings from an institutional sample

**DOI:** 10.5195/jmla.2022.1273

**Published:** 2022-01-01

**Authors:** Johanna Goldberg, Lindsay M. Boyce, Céline Soudant, Kendra Godwin

**Affiliations:** 1 goldbej2@mskcc.org, Research Informationist, Medical Library, Memorial Sloan Kettering Cancer Center, New York City, NY; 2 boycel@mskcc.org, Research Informationist, Medical Library, Memorial Sloan Kettering Cancer Center, New York City, NY; 3 soudantc@mskcc.org, Research Informationist, Medical Library, Memorial Sloan Kettering Cancer Center, New York City, NY; 4 godwink@mskcc.org, Research Informationist, Medical Library, Memorial Sloan Kettering Cancer Center, New York City, NY

**Keywords:** systematic review, meta-analysis, author instructions, journal requirements, research methodology, publishing

## Abstract

**Objectives:**

Systematic reviews and meta-analyses (SRs/MAs) are designed to be rigorous research methodologies that synthesize information and inform practice. An increase in their publication runs parallel to quality concerns and a movement toward standards to improve reporting and methodology. With the goal of informing the guidance librarians provide to SR/MA teams, this study assesses online journal author guidelines from an institutional sample to determine whether these author guidelines address SR/MA methodological quality.

**Methods:**

A Web of Science Core Collection (Clarivate) search identified SRs/MAs published in 2014–2019 by authors affiliated with a single institution. The AMSTAR 2 checklist was used to develop an assessment tool of closed questions specific to measures for SR/MA methodological quality in author guidelines, with questions added about author guidelines in general. Multiple reviewers completed the assessment.

**Results:**

The author guidelines of 141 journals were evaluated. Less than 20% addressed at least one of the assessed measures specific to SR/MA methodological quality. There was wide variation in author guidelines between journals from the same publisher apart from the American Medical Association, which consistently offered in-depth author guidelines. Normalized Eigenfactor and Article Influence Scores did not indicate author guideline breadth.

**Conclusions:**

Most author guidelines in the institutional sample did not address SR/MA methodological quality. When consulting with teams embarking on SRs/MAs, librarians should not expect author guidelines to provide details about the requirements of the target journals. Librarians should advise teams to follow established SR/MA standards, contact journal staff, and review SRs/MAs previously published in the journal.

## INTRODUCTION

Systematic reviews and meta-analyses (SRs/MAs) are rigorous research methodologies that collect information on a focused topic through a transparent and reproducible process. The goal of SRs/MAs is to synthesize the evidence to reach conclusions that inform evidence-based decision making, with MAs including statistical analysis. In evidence-based medicine, SRs/MAs are frequently placed at the top of the hierarchy of evidence and given more weight as a result [1]. As the number of published SRs/MAs has increased, however, their quality has been questioned. Halevi and Pinotti found an exponential growth in SR publications beginning in 1994, accompanied by topic saturation and a decline in quality and utility [2].

A rise in SR/MA popularity also resulted in the development of standards for SR/MA reporting and methodology. The earliest efforts date back to at least 1991, with the validation of the Overview Quality Assessment Questionnaire (OQAQ) [3]. Since then, additional tools have been developed to improve reporting quality, like Quality of Reporting of Meta-analyses (QUOROM), Preferred Reporting Items for Systematic Reviews and Meta-Analyses (PRISMA), and Meta-analysis of Observational Studies in Epidemiology (MOOSE). Other tools address methodological quality, like A Measurement Tool to Assess Systematic Reviews (AMSTAR), R-AMSTAR, and AMSTAR 2 [4–9].

Numerous studies have investigated the quality of SRs/MAs, many taking a specific interest in trends in the journals in which they are published [10–19]. Nascimento et al. found that a journal's reporting standard endorsement did not correlate with higher quality SR/MA methodology, and Riado Minguez found a lack of compliance to PRISMA and AMSTAR in anesthesiology journals, even among journals that endorsed PRISMA [20,21]. Polkki et al. reported wide variation in methodological quality in high-impact nursing journals [22]. However, very little has been published on methodological requirements in author guidelines. In a letter to the editor, Butler et al. assessed whether the top fifty anesthesiology and critical care medicine journals' author guidelines mentioned SR reporting standards, risk of bias assessment, registered protocols, quality appraisal, and use of Cochrane guidelines. Only the journal that published the letter included all five elements in its author guidelines [23]. Journals alternately refer to author guidelines as instructions, information, requirements, and guidelines; hereafter we refer to them as guidelines.

Medical librarians with a formal or informal systematic review service are tasked with teaching best practices to members of their institution interested in embarking on SR/MA projects. Often, these researchers approach the library knowing very little about the SR/MA process. At our institution, it is common practice to advise researchers publishing in diverse health sciences disciplines to review the author guidelines of their target journals at the start of their projects. Researchers frequently ask about protocol registration, reporting standards, or other requirements, and endorsed methods may differ between journals.

This recommendation to review author guidelines emerged from anecdotal evidence. To better inform librarian knowledge and practice going forward, and consequently improve the output of researchers, we assessed the publicly available online author guidelines of journals where authors affiliated with our institution recently published SRs/MAs to determine the extent to which they address SR/MA methodological quality.

## METHODS

In May 2020, we searched the Web of Science Core Collection 1945-present (Clarivate) for SRs/MAs cowritten by authors affiliated with the institution served by our library and published between 2014 and 2019. The Web of Science Core Collection included Science Citation Index Expanded (1945-present), Social Sciences Citation Index (2005-present), Arts & Humanities Citation Index (2005-present), Conference Proceedings Citation Index-Science (1994-present), Conference Proceedings Citation Index-Social Science & Humanities (1994-present), Book Citation Index-Science (2005-present), Book Citation Index-Social Sciences & Humanities (2005-present), Emerging Sources Citation Index (2015-present), Current Chemical Reactions (1985-present), and Index Chemicus (1993-present).

This institutional sample provided us with a cross-section of the medical literature that reflected the six most recent and complete years of SR/MA publishing by our user base at the time of data collection. While our institution has a primary research focus, researchers publish in health sciences journals that vary significantly by discipline. We chose to use an institutional sample to inform our work and the work of librarians at institutions where researchers publish in diverse medical fields. Web of Science offers an organization-enhanced search field that groups together variants of an institution's name, which we used to search for our institution in combination with terms for SRs/MAs. The search was adapted from the search strategy used to create the PubMed systematic reviews filter [24]. The search strategy was TI=(((systematic OR Cochrane) NEAR/3 review) OR meta-analys* OR metaanalys*) AND OG=(Memorial Sloan Kettering Cancer Center). We compiled a list of unique journals from these search results. One reviewer screened the titles returned by the search to confirm at least one citation from each journal on the list was published as an SR/MA. Any journal without an associated SR/MA was excluded from the analysis. This produced a final set of journals in which institution-affiliated authors published SRs/MAs within the given time frame.

We used Clarivate Analytics' Journal Citation Reports to capture the publisher and the 2019 Normalized Eigenfactor and 2019 Article Influence Score of each journal. Normalized Eigenfactor is a measure of a journal's scientific importance based on the number of times its articles published in the last five years were cited in a specific year. Journals with a Normalized Eigenfactor of 2.00 have twice the influence of the average journal included in Journal Citation Reports [25]. Article Influence Score is a measure of the average influence of a journal's articles in the first five years postpublication. The mean Article Influence Score in Journal Citation Reports is 1.00 [25]. Unlike journal impact factor, these two related measures adjust for citation differences across disciplines, allowing for better comparisons across fields [25].

We verified the publisher information during data collection and mapped each journal imprint or house to the largest corporate publisher entity possible (e.g., mapping both Springer and Nature to Springer Nature). We wanted to group publishers to note whether a publisher was an indicator of the breadth of the author guidelines. Journal data was saved and then collected on an Excel spreadsheet.

We developed our assessment criteria for author guidelines using AMSTAR 2, which is a widely used quality assessment tool for SRs [6]. We considered following other measures of methodological quality, like the Cochrane Handbook, Methodological Expectations of Cochrane Intervention Reviews (MECIR) Standards, the JBI Critical Appraisal Checklist, and Risk of Bias in Systematic Reviews (ROBIS), but chose AMSTAR 2 because it was the most readily applicable to journal requirements and not just to SRs/MAs themselves (e.g., the methods used in evidence synthesis to include or exclude individual studies) or to SRs/MAs sponsored by a specific organization [26–29].

Our goal was to assess author guidelines to see if they addressed SR/MA methodological quality, ultimately to inform the guidance and education librarians provide their SR/MA teams. Accordingly, we did not use the AMSTAR 2 checklist in its entirety but selected and adapted AMSTAR 2 questions about the major steps of SRs/MAs that librarians advise researchers on or address during SR/MA consultations. These included defining the research question, writing and registering a protocol, searching the literature, screening the results, assessing risk of bias, and data collection. We excluded questions that only applied to MAs, as these are typically the purview of statisticians. We also excluded questions that could not be applied to author guidelines, like examining the adequacy of descriptions of included studies or result heterogeneity.

To this targeted list of methodology questions, we added several data points to explore if and how author guidelines present SRs/MAs. These included whether the author guidelines mention SRs/MAs at all, if they have a section focused on SRs/MAs, if they require the use of a reporting standard like PRISMA (which includes requirements like providing the full search strategies for the SR/MA), and if they recommend or require a librarian or expert searcher to conduct the search, as librarian involvement has been correlated with higher SR/MA quality [30].

When author guidelines did not mention SRs/MAs, one reviewer searched PubMed (PubMed.gov) using the journal's abbreviation in the journal field, then limited the results by year to 2019–2020 and selected Systematic Review and Meta-Analysis under Article Type to determine whether the journal had published a recent SR/MA. When the journal was not currently indexed in MEDLINE, the reviewer searched Web of Science Core Collection 1945-present (Clarivate) using the journal's International Standard Serial Number (ISSN), limited the results to 2019–2020, and screened the results for articles with “systematic review” or “meta-analysis” in the titles. Journals that had not published an SR/MA within the given time frame were excluded from the analysis.

Two reviewers independently assessed the author guidelines of each journal in August 2020, coding each item as “yes” or “no” based on whether it was clearly mentioned in the author guidelines. For questions asking whether author guidelines required a specific methodology, like searching multiple databases or having two or more authors screen or extract data, language indicating that these methods were optional were coded as “no” responses. Conflicts were resolved by a second pair of reviewers through their consensus. After collecting the data, we analyzed it based on the number of elements included in the author guidelines, defining that number as an author guideline score. While AMSTAR 2 was not designed to give quality scores to SRs/MAs [6], we felt this application provided a way to more easily compare author guidelines.

We looked for patterns by author guideline score, Normalized Eigenfactor, and Article Influence Score. We classified the journals into two groups according to author guideline score (≥8 and <8), Normalized Eigenfactor (≥2.00 and <2.00), and Article Influence Score (≥1.00 and <1.00). We also looked for patterns between author guideline score and publisher.

## RESULTS

Our search returned 145 unique journals. Two journals were excluded from our analysis as publications by institutional authors were not SRs/MAs. Of the remaining journals, 33% (47/143) did not mention SRs/MAs in their author guidelines. We reviewed the publication history of these journals. Two journals did not publish SRs/MAs in 2019 or 2020 and were excluded from our analysis.

**Figure 1:**
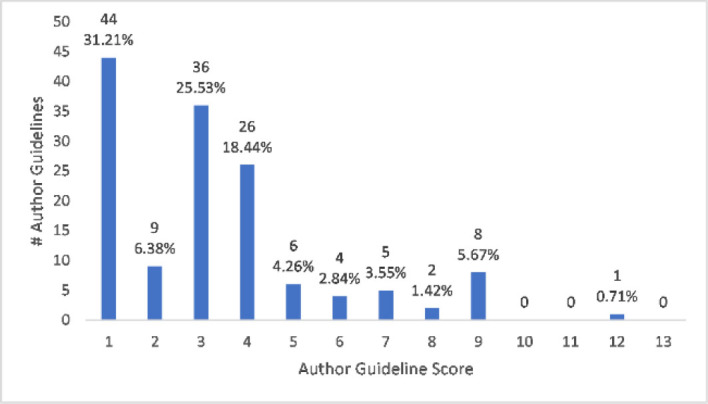
Author guideline scores by counts and percentage

More than 81% (115/141) of journals were coded as having author guidelines that produced “yes” answers to 4 or fewer questions out of 13 questions total, referred to as the author guideline score. Only around 11% (16/141) had “yes” answers to at least half of the questions, for an author guideline score of 7 or higher. About 31% (44/141) of author guidelines received an author guideline score of 1 for only instructing authors to report conflicts of interest, something not specific to SRs/MAs. See Figure 1 for a breakdown of author guideline scores by counts and percentage.

Just under 30% (42/141) of the author guidelines had a specific section, webpage, or PDF dedicated to SRs/MAs; the 99 author guidelines without this element were more difficult to navigate for pertinent SR/MA information. One author guideline did not mention SRs/MAs by name but did mention following PRISMA guidelines; 60% (85/141) of author guidelines specified an SR/MA reporting standard to follow.

### Questions specific to SR/MA methodological quality

Of the questions specific to SR/MA methodology (questions 4–12), the most frequently included item in the author guidelines was having text about writing, registering, or publishing a protocol, which 19% (27/141) of author guidelines included. However, only about 4% (6/141) of author guidelines required protocol registration: those from *BJU International, British Journal of Dermatology, Journal of Pain, Lancet, Lancet Oncology,* and *Psycho-Oncology.* Just over 8% (12/141) of author guidelines explicitly required searching in two or more databases. Of these, 7 (6 of which were from *JAMA* imprints) also recommended working with a librarian or expert searcher, an item not included in the AMSTAR 2 checklist but added to this assessment. Only 1 author guideline included requirements that two or more authors screen articles for inclusion (question 9) and extract data (question 12). More author guidelines (12%; 17/141) required authors to give inclusion/exclusion criteria and the technique used to assess risk of bias, and almost 8% (11/141) mentioned having a focused research question with specific components like population and intervention. Table 1 provides a full breakdown of responses by question.

**Table 1:** Counts and percentage of yes/no responses for each assessed author guideline question

Question	# Yes	% Yes	# No	% No
Questions not specific to SR/MA methodology (1–3)				
1. Is there text that mentions systematic reviews or meta-analyses by name?	96	68.09%	45	31.91%
2. Is there text that addresses systematic reviews with its own section, heading, webpage, or PDF?	42	29.79%	99	70.21%
3. Is there text about following PRISMA, MOOSE, or another reporting standard?	85	60.28%	56	39.72%
Questions specific to SR/MA methodology (4–12)				
4. Is there text about writing, registering, or publishing a protocol?	27	19.15%	114	80.85%
5. Is there text that protocol registration is mandatory?	6	4.26%	135	95.74%
6. Is there text about having a focused research question with specific components, like population and intervention?	11	7.80%	130	92.20%
7. Is there text about requiring searches in two or more databases?	12	8.51%	129	91.49%
8. Is there text that recommends or requires working with a librarian?	7	4.96%	134	95.04%
9. Is there text about requiring two or more authors when screening articles for inclusion?	1	0.71%	140	99.29%
10. Is there text about including inclusion and exclusion criteria?	17	12.06%	124	87.94%
11. Is there text about including the technique used to assess risk of bias?	17	12.06%	124	87.94%
12. Is there text about requiring two or more authors when extracting data?	1	0.71%	140	99.29%
Question related to all research methodology (13)				
13. Is there text that instructs the authors to report their conflicts of interest, like funding sources?	141	100.00%	0	0.00%

### Journals with the highest author guideline scores

As indicated in Figure 1, which offers a breakdown of author guideline scores in our institutional sample, only about 8% (11/141) of author guidelines had a score of 8 or higher. The journals with the highest author guideline scores are delineated in Table 2. *BJU International,* the journal with the highest scoring author guidelines, registered only one “no” answer; this was in response to having a focused research question with specific components, like population and intervention (question 6). See our supplemental data for full information on all journals and the composition of each author guideline score.

**Table 2:** Journals with the highest author guideline scores

Title	Publisher	Author guideline score (# Yes responses)
*BJU International*	John Wiley & Sons, Inc	12
*JAMA Cardiology*	American Medical Association	9
*JAMA Dermatology*	American Medical Association	9
*JAMA Internal Medicine*	American Medical Association	9
*JAMA Oncology*	American Medical Association	9
*JAMA Otolaryngology-Head & Neck Surgery*	American Medical Association	9
*JAMA-Journal of the American Medical Association*	American Medical Association	9
*Lancet*	Elsevier	9
*Lancet Oncology*	Elsevier	9
*Developmental Psychology*	American Psychological Association	8
*JAMA Network Open*	American Medical Association	8

### Author guideline scores by Normalized Eigenfactor and Article Influence Score

Seven journals in our institutional sample were not included in Journal Citation Reports and did not have a Normalized Eigenfactor assigned. Of the remaining 134 journals, 69 had a Normalized Eigenfactor above 2.00. While 9 of the 11 journals with author guideline scores of 8 or higher had a Normalized Eigenfactor above 2.00, there were also 21 journals with a Normalized Eigenfactor above 2.00 with an author guideline score of 1 or 2. By comparison, 2 journals with an author guideline score of 8 or higher were among the 65 with a Normalized Eigenfactor below 2.00, and 27 journals with author guideline scores of 1 or 2 had a Normalized Eigenfactor below 2.00.

Of the 141 journals, 129 had Article Influence Scores included in Journal Citation Reports. Of these, 73 had an Article Influence Score above 1.00. All journals with author guideline scores of 8 or above fell into this group. There were 20 journals with an Article Influence Score above 1.00 that had author guideline scores of 1 or 2. In comparison, 27 journals with an Article Influence Score below 1.00 had author guideline scores of 1 or 2.

### Author guideline scores by publisher

After mapping the 141 journals to the largest corporate publisher entity possible, we identified 25 unique publishers. There was often wide variation in author guidelines within one publisher. For example, while the top publishers by number of associated journals in our institutional sample (Elsevier, Springer Nature, Wolters Kluwer, and John Wiley & Sons) had at least one journal with an author guideline score of 7 or higher, these same publishers also had the highest number of journals with an author guideline score of 1. While the author guideline with the highest author guideline score was from a journal published by John Wiley & Sons, 38% (5/13) of their surveyed journals' author guidelines did not mention SRs/MAs. The American Medical Association was the only publisher to stand out for consistency and quality, both in number of similar and of high author guideline scores. All 7 journals from this publisher appear in the list of journals with the highest author guideline scores, noted in Table 2. Table 3 provides a breakdown of author guideline scores by publisher.

**Table 3:** Range and mean of author guideline scores by publisher

Publishers	Number of journals	Range of author guideline scores (when more than one journal)	Mean author guideline score
AME Publishing Company	3	3	3.00
American Association for Cancer Research	3	3	3.00
American Association of Neurological Surgeons	1		3.00
American College of Physicians	1		5.00
American Medical Association	7	8–9	8.85
American Psychological Association	1		8.00
American Roentgen Ray Society	1		4.00
American Society of Clinical Oncology	1		3.00
British Medical Association	1		4.00
Cambridge University Press	1		2.00
Elsevier	42	1–9	2.74
Impact Journals LLC	1		1.00
Insight Medical Publishing (iMedPub Ltd)	1		1.00
John Wiley & Sons, Inc	13	1–12	2.46
Karger	1		1.00
Oncology Nursing Society	2	1–4	2.00
Oxford University Press	6	1–5	2.33
Public Library of Science	2	5	5.00
Radiological Society of North America	1		3.00
SAGE Publishing	7	3	3.00
Springer Nature	22	1–7	3.50
Taylor & Francis Group	6	1–4	2.04
Termedia Publishing House	1		1.00
Thieme Medical Publishing Group	1		1.00
Wolters Kluwer	15	1–7	2.80

## DISCUSSION

### Author guideline inclusion of SR/MA methodological quality measures

The majority of author guidelines included in this institutional sample did not include information addressing SR/MA methodological quality, indicating that librarians and researchers should expect to seek this information from other established sources. Journals most frequently included information in their author guidelines not specific to SRs/MAs (e.g., author conflicts of interest) or not about SR/MA methodological quality (e.g., mentioning SRs/MAs or following a reporting standard). Of questions specific to SR/MA methodology, the most frequently included item was about writing, registering, or publishing a protocol (19%; 27/141). Still, only 4% (6/141) of the journals required protocol registration. The least commonly included items were requiring two or more authors to screen and to extract data, both of which were included by 1 journal (0.71%).

### Many journals do not follow established standards for author guidelines

While our finding that there is limited methodological guidance for SRs/MAs in author guidelines may not be surprising, it was unexpected that 31.9% (45/141) of the journals did not mention SRs/MAs in their author guidelines, though these journals publish SRs/MAs. The “Principles of Transparency and Best Practice in Scholarly Publishing” from the Committee on Publication Ethics (COPE), Directory of Open Access Journals (DOAJ), Open Access Scholarly Publishers Association (OASPA), and World Association of Medical Editors (WAME) states: “There should be a statement on what a journal will consider for publication” [31]. The International Committee of Medical Journal Editors (ICMJE)'s widely endorsed “Recommendations for the Conduct, Reporting, Editing, and Publication of Scholarly Work in Medical Journals” does not indicate that journals should list the types of studies they publish. The ICMJE's recommendations do encourage journals to ask authors to follow reporting guidelines [32]. The PRISMA reporting guideline was first mentioned in the 2013 version of these recommendations. In a 2011 study, Tao et al. found that about 29% of author guidelines (42/146) mentioned following PRISMA or QUOROM [33]. Our study found that 60% (85/141) of author guidelines mentioned an SR/MA reporting standard. While there is room for improvement, ICMJE's recommendations have likely played a role in this increase.

### Comparing author guidelines for SRs/MAs to author guidelines for clinical trials

Previous studies of author guidelines have shown omissions in study types beyond SRs/MAs. Several have looked specifically at whether author guidelines mention clinical trial registration requirements. In one study of hematology and oncology journals, 42.9% (99/231) of author guidelines mentioned trial registration, while 35.5% (82/231) required it [34]. Studies of author guidelines in several other disciplines have found rates of journal author guidelines mentioning clinical trial registration ranging from 43% to 86%, with the highest percentage from a sample of 21 general medical journals [35–39]. Even in study types like clinical trials, author guidelines do not always endorse best practices. In addition, studies have shown that endorsement of standards does not necessarily lead to compliance [20,21].

### Lessons for librarians

While some author guidelines in our institutional sample provided methodological guidance that could inform published SR/MA quality, and received higher author guideline scores as a result, most did not. This finding is in line with the low methodological guidance present in author guidelines for other study types, like clinical trials, for which librarians collaborate less with researchers. Librarians should not expect author guidelines to provide detail about SR/MA methodological quality. This should be considered and shared when consulting with teams embarking on SRs/MAs.

Our analysis, based on descriptive statistics, did not reveal journal attributes frequently indicative of methodologically detailed author guidelines, except for being published by the American Medical Association. One of the items not included in the AMSTAR 2 checklist that we added to our analysis—inclusion of a librarian—was infrequently mentioned, except by American Medical Association author guidelines. Otherwise, there was no relationship between publisher and author guideline score. Normalized Eigenfactor also did not indicate author guideline score. While journals with Article Influence Scores of 1.00 or more were more likely to have higher author guideline scores, a higher Article Influence Score did not necessarily indicate that these author guidelines addressed methodological quality, as measured by our questions. As it is difficult to know which journal will offer detailed SR/MA author guidelines based on its attributes alone, it remains advisable for researchers to review their target journals' author guidelines at the start of their SR/MA project, in case they provide clear expectations. This is especially important for journals with author guidelines that note requiring protocol registration.

Given the percentage of author guidelines in this institutional sample that did not mention SRs/MAs, researchers should check the publication history of their target journals and/or contact the journals' editors to see whether the journals publish SRs/MAs and the standards the journals follow. However, the onus is on the librarian and the researchers, respectively, to recommend and follow SR/MA best practices.

## LIMITATIONS

There are limitations to this analysis. The dataset only included journals in which specialized researchers from a single institution published SRs/MAs within a specific time frame. A larger, randomized sample set may have allowed for a more thorough analysis with more widely applicable findings. Questions in the author guidelines assessment tool were developed based on the AMSTAR 2 checklist but were also influenced by librarian experience with SR/MA teams, leading to potential bias in the data collected. Journal publishers and author guidelines change over time. Publicly available online author guidelines are only one indicator of a journal's standards.

## CONCLUSION

Our study of 141 author guidelines found that the majority did not address SR/MA methodological quality; 31.9% (45/141) did not mention SRs/MAs at all. Characteristics like Normalized Eigenfactor and publisher did not correspond to author guideline score, with author guidelines from American Medical Association journals as an exception to the latter. Higher Article Influence Scores were only slightly reflective of a journal's likelihood of having a higher author guideline score. While librarians may still choose to direct researchers to author guidelines, they need to be aware that the guidance these provide could be minimal. In supporting SR/MA teams, librarians should provide, demonstrate, and encourage the use of established best practices, even when these best practices are left out of author guidelines.

## Data Availability

Data associated with this article are available in the Open Science Framework at https://osf.io/fwcxj/.
